# Serum Based Diagnosis of Asthma Using Raman Spectroscopy: An Early Phase Pilot Study

**DOI:** 10.1371/journal.pone.0078921

**Published:** 2013-11-08

**Authors:** Aditi Sahu, Krishna Dalal, Sarla Naglot, Parveen Aggarwal, C. Murali Krishna

**Affiliations:** 1 KS-04, Chilakapati Laboratory, ACTREC, Kharghar, Navi Mumbai, India; 2 Department of Biophysics, All India Institute of Medical Sciences, Ansari Nagar, New Delhi, India; 3 Department of Emergency Medicine, All India Institute of Medical Sciences, Ansari Nagar, New Delhi, India; Aligarh Muslim University, India

## Abstract

The currently prescribed tests for asthma diagnosis require compulsory patient compliance, and are usually not sensitive to mild asthma. Development of an objective test using minimally invasive samples for diagnosing and monitoring of the response of asthma may help better management of the disease. Raman spectroscopy (RS) has previously shown potential in several biomedical applications, including pharmacology and forensics. In this study, we have explored the feasibility of detecting asthma and determining treatment response in asthma patients, through RS of serum. Serum samples from 44 asthma subjects of different grades (mild, moderate, treated severe and untreated severe) and from 15 reference subjects were subjected to Raman spectroscopic analysis and YKL-40 measurements. The force expiratory volume in 1 second (FEV1) values were used as gold standard and the serum YKL-40 levels were used as an additional parameter for diagnosing the different grades of asthma. For spectral acquisition, serum was placed on a calcium fluoride (CaF_2_) window and spectra were recorded using Raman microprobe. Mean and difference spectra comparisons indicated significant differences between asthma and reference spectra. Differences like changes in protein structure, increase in DNA specific bands and increased glycosaminoglycans-like features were more prominent with increase in asthma severity. Multivariate tools using Principal-component-analysis (PCA) and Principal-component based-linear-discriminant analysis (PC-LDA) followed by Leave-one-out-cross-validation (LOOCV), were employed for data analyses. PCA and PC-LDA results indicate separation of all asthma groups from the reference group, with minor overlap (19.4%) between reference and mild groups. No overlap was observed between the treated severe and untreated severe groups, indicating that patient response to treatment could be determined. Overall promising results were obtained, and a large scale validation study on random subjects is warranted before the routine clinical usage of this technique.

## Introduction

Asthma is a chronic inflammatory disorder of the airways characterized by airway hyper-responsiveness (AHR) and reversible airflow obstruction that fluctuates over time [1]. Airway obstruction and allergic inflammation during the disease occur due to release of IgE and pro-inflammatory cytokines such as interleukin(IL)-4, IL-5, IL-9, and IL-13 by T helper cell type 2 (Th2) and other immune effector cells producing toxic inflammatory molecules that ultimately elicit obstruction [2,3]. According to WHO estimates, 300 million people suffer from Asthma. In India, an estimated 57,000 deaths were attributed to Asthma in 2004 [4,5]. Earlier, asthma was considered a single disease entity, but in recent times multiple sub-phenotypes that differ in clinical severity, pathological findings, response to therapy, and long-term outcome have been discovered [6-8]. Based on clinical parameters, patients are assigned to distinct categories (mild, moderate, severe or very severe) that allow optimal medical decisions on treatment and prognosis to be made for individual phenotypes.

Current diagnosis of asthma is based on a history of wheeze, shortness of breath, and cough, which are variable in severity and over time [9]. New international guidelines recommend the measurement of serial peak expiratory flows, spirometry or bronchoprovocation to confirm the diagnosis of asthma [9-11]. However, these are primarily based on demonstrating abnormal airway physiology, which may not always be present in mild asthma, leading to a decreased sensitivity. Some other associated disadvantages including the need of patient compliance, high dependence on patient cooperation and effort, and the need for patient understanding reduce the overall diagnostic utility of these tests [12,13]. In a clinical setting, diagnosis accomplished in a non-invasive or minimally invasive manner, for example, using body fluids is highly desirable. Body ﬂuids have garnered importance for biomarker identiﬁcation, because of the advantages in accessibility, less invasive procedure, low cost, and multiple sampling for monitoring the disease development [14]. Several studies investigating biomarkers in serum, mainly the molecules involved in the interplay between the host inflammatory response and allergen, are underway. Recent reports indicate the potential utility of YKL-40, a chitinase like protein belonging to 18 glycosyl hydrolase family and expressed in inflammatory conditions, as a possible biomarker of asthma [15-17]. 

Biological ﬂuids are also being widely investigated with the use of molecular spectroscopy for clinical diagnosis of various diseases - degenerative, cancerous, and others. The methods newly introduced in the last decade for such investigations are Raman-, infrared-, and ﬂuorescence- spectroscopy. Fluorescence spectroscopy for oral and breast cancer detection has been reported [18,19]. Several IR spectroscopy studies on blood have shown its potential in detecting various diseases including Alzheimer’s, scrapie, sickle cell anaemia and several cancers [20-25]. Raman spectroscopy (RS), a vibrational spectroscopic method based on inelastic scattering of light, has been widely used for qualitative characterization of biological tissues [26-28], for diagnosis of periodontitis and lung cancer from saliva [29,30], breast cancers [31], atherosclerosis [32] and also for several cancers using serum [33-36]. For quantitative analysis, RS has been employed for determining glucose and other analytes in blood [37,38]. RS of body fluids has also shown potential in a multitude of biomedical applications, including pharmacological and forensic applications [39-45]. Thus, the RS of body fluids could be used to monitor metabolic changes occurring with disease. In light of need for better diagnostic tools with attributes like rapidity, objectivity and the use of minimally invasive samples, RS could serve useful for detection of asthma. Thus, this pilot study was carried out to explore the potential applicability of RS in detecting disease-related perturbations in serum of mild, moderate and severe asthma as well as reference subjects. The force expiratory volume in 1 second (FEV1) values were used as gold standard to verify the diagnosis of active subjects and to classify the different grades of asthma. The serum YKL-40 level was measured and the estimated levels were used as an additional parameter to confirm the clinical status of all subjects. 

## Materials and Methods

### 1: Subject recruitment

#### 1.1: Ethics statement

Approval to conduct the study was issued by the human ethics committee of All India Institute of Medical Sciences (AIIMS), New Delhi. A written informed consent was taken from each participant following the ethical norms of the institute.  Access to the data was restricted to the investigators and was not available to the public.

#### 1.2: Location of the study and study subjects

The subjects were screened and selected in the study from the Out Patient services of Department of Medicine, AIIMS, New Delhi following the Asthma criteria. For reference group, 15 subjects (age and gender matched) were chosen from patients’ relatives, friends and colleagues. Raman spectroscopic studies using serum of these subjects were carried out at Advanced Centre for Training, Research and Education in Cancer (ACTREC), Navi Mumbai, India. Serum YKL-40 levels were estimated at AIIMS, New Delhi. 

#### 1.3: Subject screening

A sample size of 44 subjects was included in the active arm of the study using gold standard diagnostic tests like Pulmonary Function Test for FEV1 values (as per Global Initiative for Asthma [GINA] guidelines), chest X-ray and complete blood count (CBC). The asthmatic conditions were further classified into 4 different categories viz., mild (n=12), moderate (n=12), untreated severe (n=10) and treated severe (n=10) cases, following the diagnostic criteria laid down by GINA guidelines. The ongoing pharmacological treatment for all the active group subjects comprised of either alone or a combination of the following medications viz., short acting β2 agonist (SABA), Long acting β2 agonist (LABA), inhaled corticosteroids (ICS) and Methylxanthines. And this treatment was not suspended for any of the sub-groups prior to sample collection. The blood samples were collected at the time of admission and before administering any additional treatment in the respective outpatient and emergency departments for the mild, moderate and untreated severe groups, respectively. The treated severe group was comprised of patients who required additional treatment due to medical urgency. These patients were administered with Salbutamol (albuterol) and Ipratropium bromide nebulization along with intravenous corticosteroids (100mg hydrocortisone 6 hourly). The blood samples were collected from these patients just before their discharge from the emergency ward when they showed significant improvement in their FEV1 values (approximately 3-10 hrs after the initiation of drugs required for severity). The reference group was constituted by healthy subjects (n=15) chosen from patients’ own relations, friends and colleagues. These subjects were declared ‘control’ (reference group) without any clinical history of disease as reported by themselves. 

#### 1.4: Serum separation

A quantity of 5 ml blood was collected from each subject in a micro-centrifuge tube (Axygen Inc.,USA) with the help of a sterile injection after informed and written consent. Samples were placed standing for 30 minutes to allow clot formation and then centrifuged at 3500 rpm for 10 minutes. After removing the fat body with the help of a microtip, samples were centrifuged again at 3500 rpm for 10 minutes. The obtained serum was aliquoted in different tubes and stored at -80°C till use. One aliquot of each sample was transported to ACTREC, Mumbai under cold conditions (4°C) for Raman spectroscopic analysis and other set of samples was subjected to YKL-40 protein quantification at AIIMS. All samples were subjected to the identical pre-treatment procedure which included initial storage at -20°C, transportation in cold packs at 4°C, and long term storage at -80° on arrival. After 24 hours, samples were allowed to thaw passively, following which Raman spectra were acquired. 

### 2: Estimation of serum YKL-40

YKL-40 levels were detected in the serum of 44 patients (with 4 subgroups, viz., mild, moderate, untreated severe and treated severe) and 15 reference subjects by surface plasmon resonance (SPR) analysis (BIAcore 2000; Pharmacia Biosciences) after immobilizing anti- YKL-40 antibody [gp-39 (N-16):Sc-30464] procured from Santa Cruz Biotechnology Inc. on the sensor chip of the instrument. The results of this study were statistically analyzed using Stata SE 11.1 software (Stata Corp, TX, USA). Results were summarized as mean±SD or median (range) depending on the nature of the distributions of the data. Since the data was not normally distributed, comparison between groups was performed by Kruskal–Wallis test followed by multiple comparison using Bonferroni correction. A p-value of less than 0.05 was considered to be statistically significant in this study. The level elevation of YKL-40 protein level in asthma (active group) subjects with respect to the reference group was calculated using equation 1 which is stated as follows: 

$$\%\;elevation\;of\;serum\;YKL-40=\frac{active\;group\;level}{reference\;group\;level}\times 100$$

In equation 1, ‘active group level’ stands for the median serum YKL-40 protein level in the individual category of asthma patients, such as mild, moderate, treated severe and untreated severe. The ‘reference group level’ stands for the median serum YKL-40 protein level of the healthy individuals. A positive sign indicates elevation in YKL-40 protein levels.

### 3: Raman spectroscopy

#### 3.1: Sample preparation for Raman spectroscopy

Serum samples were transported to ACTREC under cold conditions to avoid degradation of serum constituents. Upon arrival, samples were immediately transferred to –80°C, and removed from cold storage only immediately prior to Raman analysis. Spectra were recorded after passive thawing of the frozen serum samples. The serum samples from different groups were run randomly.

#### 3.2: Spectral acquisition

**Figure 1:**
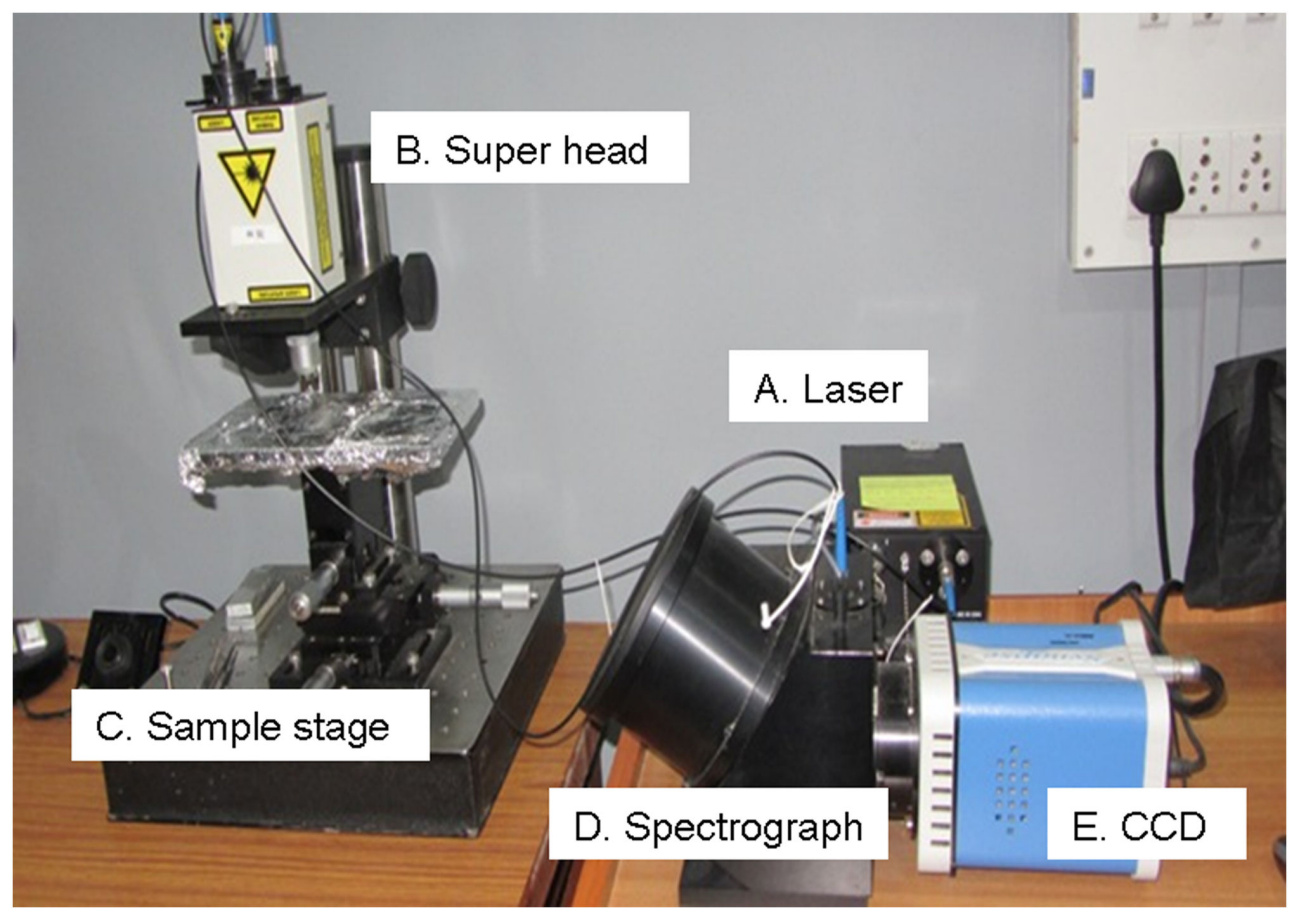
Raman instrumentation for *ex* *vivo* applications. A. Laser: excitation source, λ_785 nm_, B. Superhead: optical assembly that couples excitation and detection elements, C. Sample stage: assembly to place *ex* *vivo* samples, D. Spectrograph: E. CCD.

After passive thawing, samples were subjected to Raman spectroscopy by placing 30 μl serum sample on calcium fluoride (CaF_2_) window and recording spectra using Fiber Optic Raman microprobe (Horiba-Jobin-Yvon, France). This Raman system consists of laser (785 nm, Process Instruments) as an excitation source and HE 785 spectrograph (Horiba-Jobin-Yvon, France) coupled with CCD (Synapse, Horiba-Jobin-Yvon) as dispersion and detection elements respectively. Optical filtering of unwanted noise, including Rayleigh signals, is accomplished through ‘Superhead’, the other component of the system. Optical fibers were employed to carry the incident light from the excitation source to the sample and also to collect the Raman scattered light from the sample to the detection system. Raman microprobe was assembled by coupling a 40X microscopic objective (Nikon, Japan) to the superhead (Figure 1). Spectral acquisition details were: Excitation wavelength (λ_ex_) = 785 nm, laser power = 40 mW. Spectra were integrated for 10 seconds and averaged over 6 accumulations. On an average, 8 spectra were recorded from each sample to generate a total of 396 spectra under 5 groups, 98 spectra from 15 reference group samples, 82 from 12 mild asthma samples, 80 spectra from 12 moderate asthma samples, 70 from 10 treated severe cases and 66 spectra from 10 untreated severe asthma samples, respectively. 

#### 3.3: Spectral pre-processing

Raman spectra from all serum samples were corrected for Charged coupled device (CCD) response with a National Institute of Standards and Technology (NIST) certified Standard Reference Material 2241 (SRM 2241) followed by the subtraction of background signals from optical elements and substrate. To remove interference of the slow moving background, first derivatives of spectra (Savitzky-Golay method and window size 3) were computed [46,47]. Spectra were interpolated in 800-1800 cm^-1^ region, vector-normalized and used as input for multivariate analysis.

#### 3.4: Multivariate analysis

Vector normalized first derivatives of spectra were subjected to multivariate unsupervised principal component analysis (PCA) and supervised Principal Component-Linear Discriminant Analysis (PC-LDA). PCA is a routinely used method for data compression and visualization. It describes data variance by identifying a new set of orthogonal features, called as principal components (PCs) or factors. For visual discrimination, we project each of the spectra in the newly formed co-ordinate space of selected PCs. While PCA aims to identify features that represent variance among complete data, LDA provides data classification based on an optimized criterion which is aimed for better class separability. In LDA, the classification criterion is identified using the scatter measure of within class and between class variance. LDA can be used in companion with PCA to increase efficiency of classification. For this, PCA scores obtained using a set of significant PCs with maximum variance amongst data are used as input data for LDA based classification. The advantage of doing this is to remove or minimize noise from the data and concentrate on variables important for classification. In our analysis, significant principal components (p<0.05) were selected as input for LDA. In order to avoid over-fitting of the data, as a thumb rule, total number of factors selected for analysis were less than half the number of the spectra in the smallest group [48]. LDA models were validated by Leave-one-out cross-validation (LOOCV). LOOCV is a type of rotation estimation used mainly for smaller datasets i.e. a technique useful for assessing performance of a predictive model with a hypothetical validation set when an explicit validation set is not available. Algorithms for these analyses were implemented in MATLAB (Mathworks Inc., USA) based software using in-house codes [49]. 

Average spectra were computed from the background subtracted spectra prior to derivatization for each class, by averaging Y-axis variations keeping X-axis constant for each class, and baseline corrected by fitting a fifth order polynomial function. These baseline corrected spectra were used for computing mean and standard deviation spectra to illustrate the intra-group heterogeneity. Difference spectra were also calculated by subtracting average spectra of reference group from different grades of asthma. 

## Results and Discussion

This early phase pilot study was undertaken to explore the possibility of RS based diagnosis of asthma using a minimally invasive sample like serum. Such an approach could serve as an objective method for asthma diagnosis for all patients (irrespective of age) and could also enable “distance diagnosis” where the samples could be transported to a centralized facility for analyses.

### YKL-40 and demographic data

**Figure 2:**
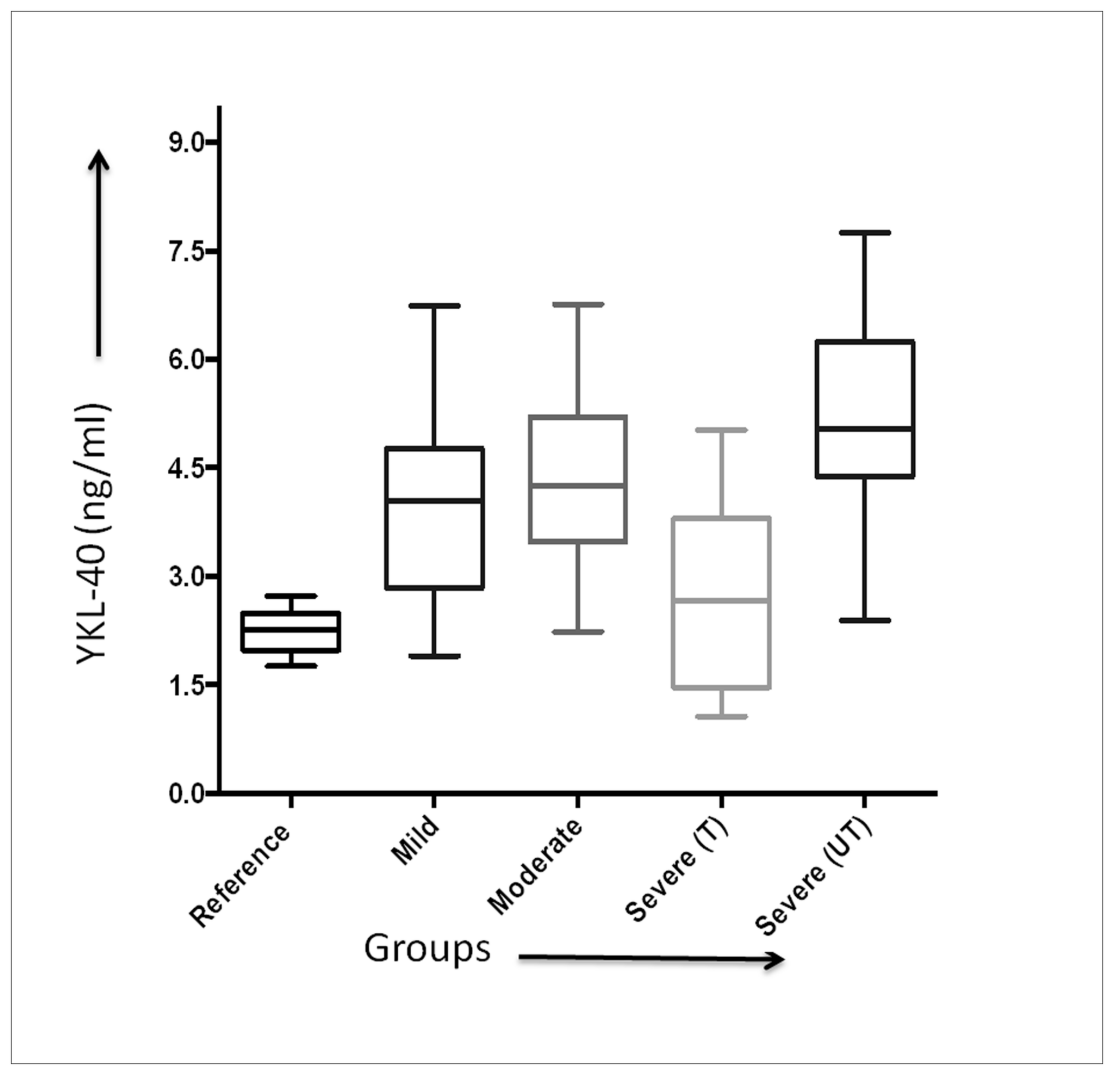
Box-and-Whisker plot exhibiting the relationships of the measured YKL-40 protein levels with reference and active groups (with different sub-groups).

The measured YKL-40 protein levels together with the demographic data for each group are summarized in Table 1. The Box-and-Whisker Plot relating the variations of YKL-40 levels with different groups is shown in Figure 2. Results indicated a definite difference between mild, moderate and untreated severe groups as compared to reference individuals, with statistically high significances (p<0.001). No statistically significant difference in YKL-40 levels was observed between the controls and the treated severe group (p-value = 0.2918). The elevations of YKL-40 protein levels in different categories of asthma compared with the control (reference) group were found to be distinct and are stated as follows: mild (79.2%), moderate (88.0%), untreated severe (122.5%) and treated severe (17.7%).

**Table 1:** Summary of subject accrual and subject characteristics.

**Variables**	**Control group**	**Active group (n = 44)**			
	**(n = 15)**	**Mild (n = 12)**	**Moderate (n = 12)**	**Severe (Untreated) (n = 10)**	**Severe (Treated) (n = 10)**
**Age (in yrs): Median (range)**	28 (22-38)	29(25-40)	33.5 (26-52)	35(26-95)	37.5(18-98)
**Gender (Male : Female)**	2 : 1	1 : 3	7 : 5	1 : 1	2 : 3
**n_smokers_ : n_never smokers_**	0 : 15	1 : 5	1 : 1	0 : 10	0 : 10
**Family history of asthma: n_with_ : n_without_**	0 : 15	3 : 1	5 : 7	1 : 9	3 : 2
**Duration of asthma in years: median (range)**		8.5(3-28)	8(2-35)	6(4-21)	5.5(1-25)
**FEV1 values (% predicted) (Mean ± SD)**		90.7 ±5.5	69.8±3.9	36.6± 14.5	70.5±23.2
**YKL-40 Levels (ng/ml)L: Median (range)**	2.3(1.8 - 2.7)	4.1(1.9–6.7)	4.3(2.2-6.8)	5.0(2.4-7.8)	2.7( 1.1-5.0)
**p-value: (individual group compared with reference group)**		0.0004	0.0001	0.0001	0.2918

### Spectral features

**Figure 3:**
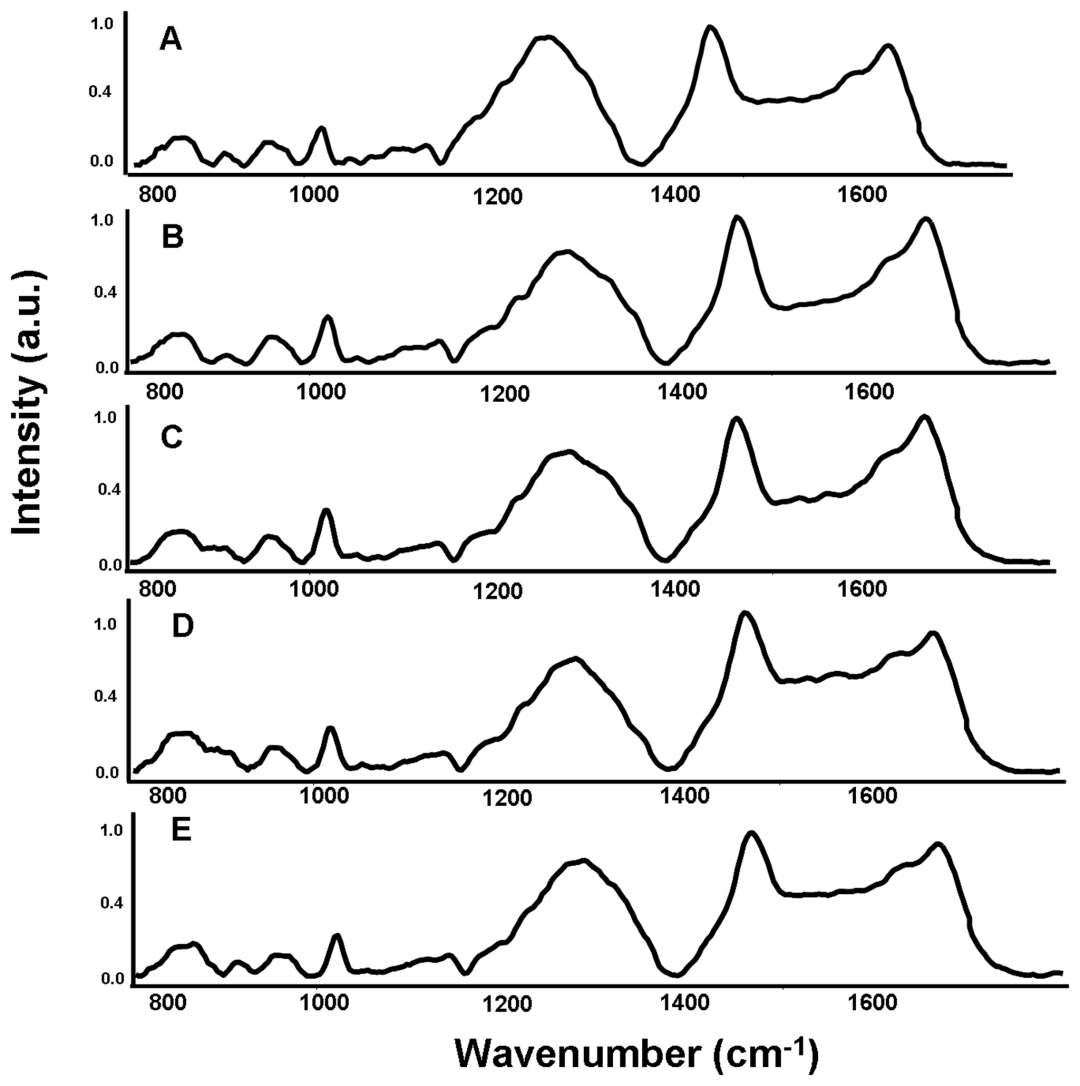
Mean Raman spectrum of reference and different asthma grades. A. Reference, B. Mild asthma, C. Moderate asthma, D. Treated severe asthma, E. Untreated severe asthma.

**Figure 4:**
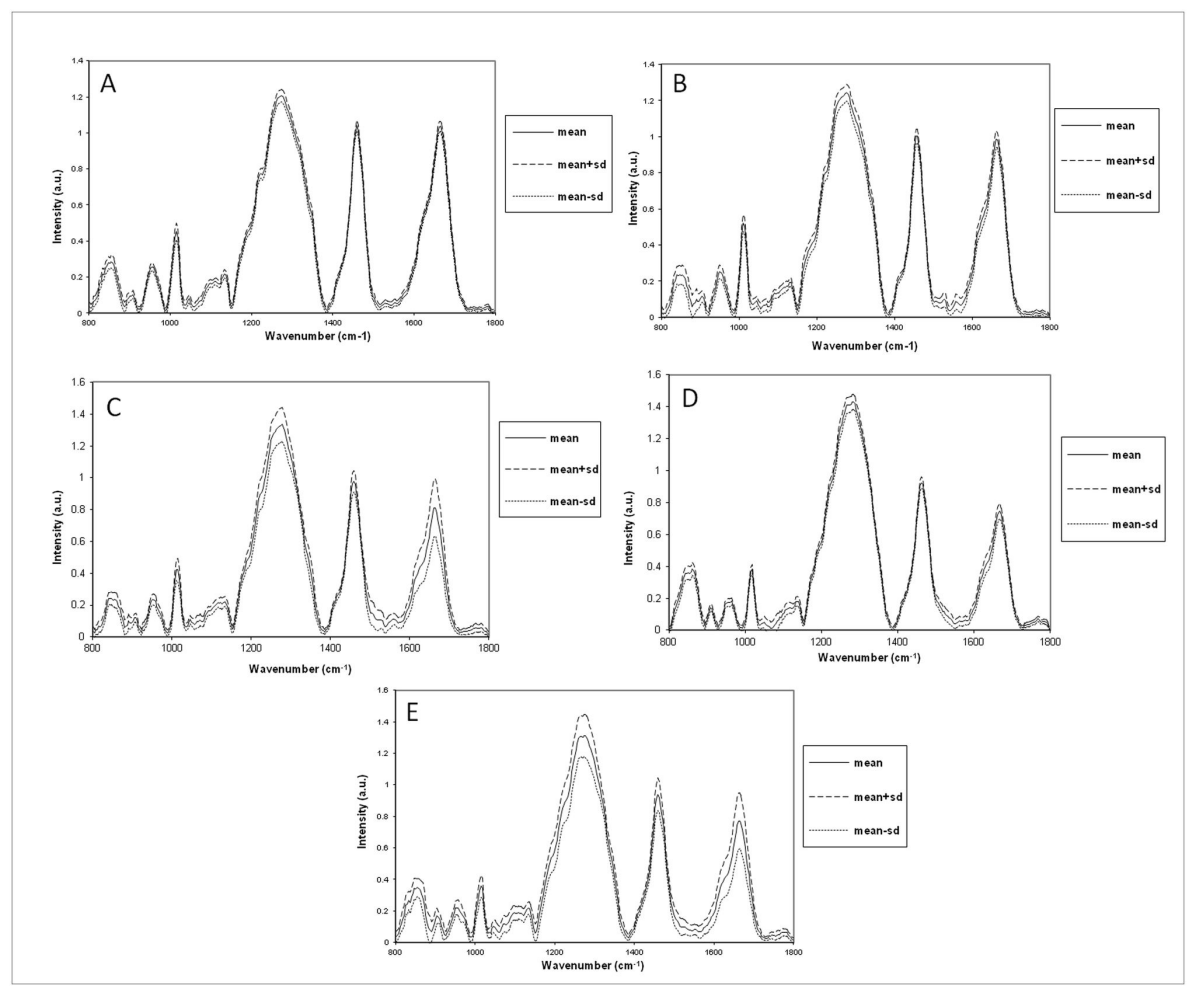
Mean and standard deviation of reference and different asthma grades. A. Reference, B. Mild asthma, C. Moderate asthma, D. Treated severe asthma, E. Untreated severe asthma.

On comparison of the normalized average spectra from reference and active groups, prominent spectral differences were observed in the amide I region, Phe-ring breathing, δCH_2_ deformation and DNA specific bands which may indicate changes in the secondary structure and in the relative concentration of proteins and DNA in these groups, as shown in Figure 3A-E. The mean and standard deviation spectra were computed for each group and heterogeneity in the spectra were limited to minor intensity related variations, as shown in Figure 4A-E. 

**Figure 5:**
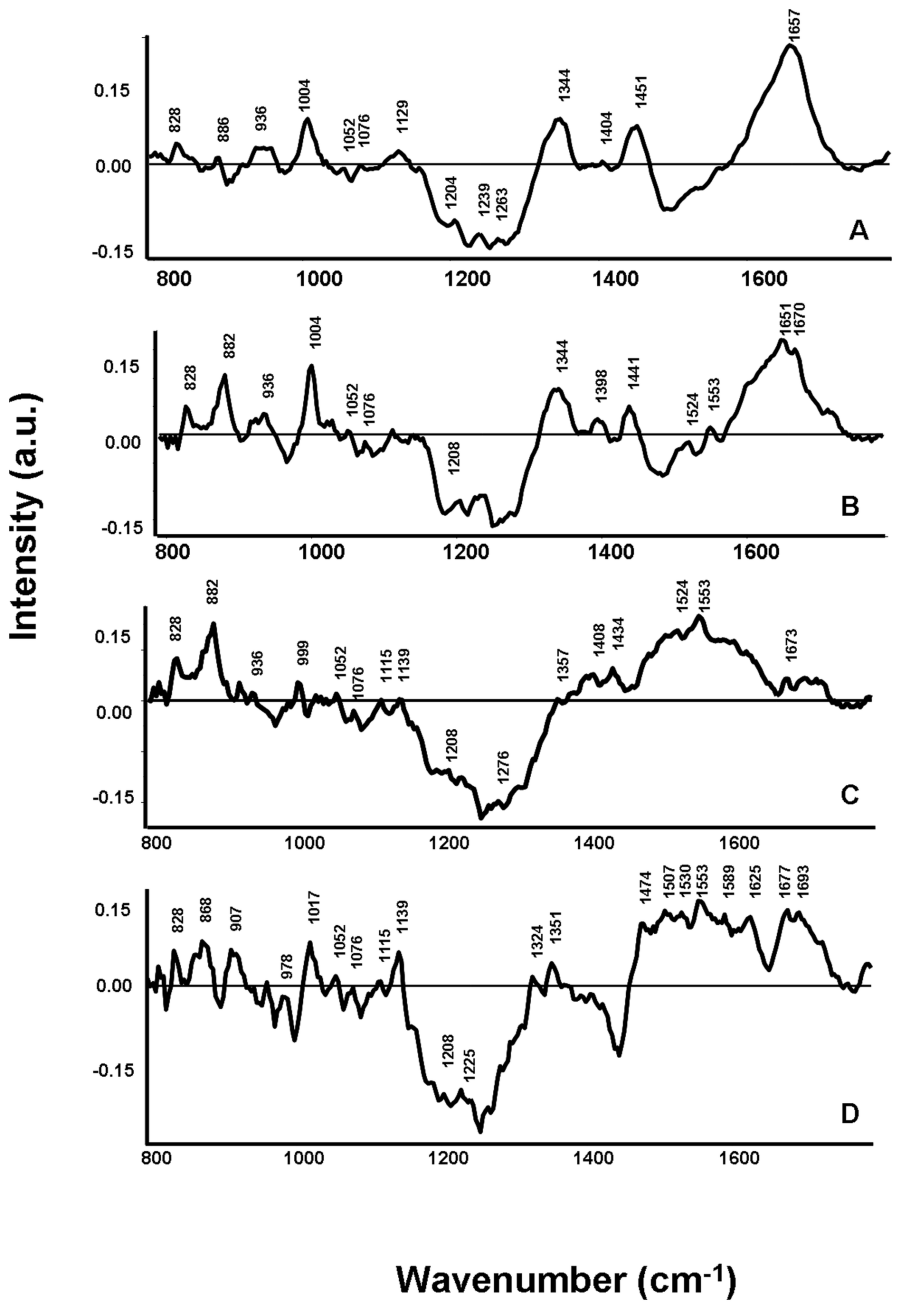
Difference spectra of the asthma groups. Difference spectra were computed by subtracting average reference spectrum from average spectrum of each asthma grade: mild, moderate, treated severe and untreated severe. A. Mild asthma-reference, B. Moderate asthma-reference, C. Treated severe asthma- reference, D. Untreated severe asthma-reference.

Difference spectra were computed to bring out spectral differences. Subtraction of mean spectra is one of the conventional ways of looking at spectral differences, which can provide differences over selected spectral range and understanding of the moieties that may have been modified. Difference spectra were obtained by subtracting normalized average spectrum of reference group from all asthma groups: mild, moderate, treated severe and untreated severe (Figure 5A-D). All positive peaks belong to the pathological groups (mild, moderate, treated severe and untreated severe asthma), while all negative peaks are the features of reference group. Difference in mild and moderate asthma grades spectra revealed positive bands at ~1004 cm^-1^, 1450 cm^-1^, 1660 cm^-1^, 1340 cm^-1^ and ~830 cm^-1^ and negative peaks at ~1260 cm^-1^, ~1076 cm^-1^, 1495 cm^-1^and 1525 cm^-1^ and 1560 cm^-1^. These peaks may be the indicative of molecules like proteins, plasma free amino acids and DNA with relatively higher concentrations persisting in the pathological (asthmatic) groups’ sera [50].

The difference spectrum of the treated severe asthma group indicated additional positive peaks in the 800-1000 cm^-1^ and 1400-1600 cm^-1^ as compared to mild and moderate difference spectra. Similarly, in the untreated severe difference spectra, increased number of positive peaks could be observed in the same region. The bands observed could be attributed to the enhanced immune hyper-responsiveness in severe asthmatic conditions that might lead to a higher secretion of histamines, leukotrienes and prostaglandins and the increased deposition of glycosaminoglycans (like galactosamine, glucoronic acid and glucosamine) in the airways [51-54]. Sufficient literature for precise spectral and biochemical correlation was not available. Biochemical assays (for qualitative and quantitative determination of implicated biomolecules) will be undertaken to achieve better correlation. 

### Multivariate data analysis

To explore the feasibility of classifying the different grades of asthma serum from reference group, multivariate tools PCA and PC-LDA were used. The results of PCA were depicted as scatter plots, generated by plotting various combinations of scores of factors. The results of PC-LDA were also depicted as scatter plots, and furthermore in the form of confusion matrix. All diagonal elements represent true positive predictions and ex-diagonal elements indicate false positive predictions. The confusion matrix was generated to understand separation between the groups obtained by accounting for contributions of all factors selected for analyses. 

**Figure 6:**
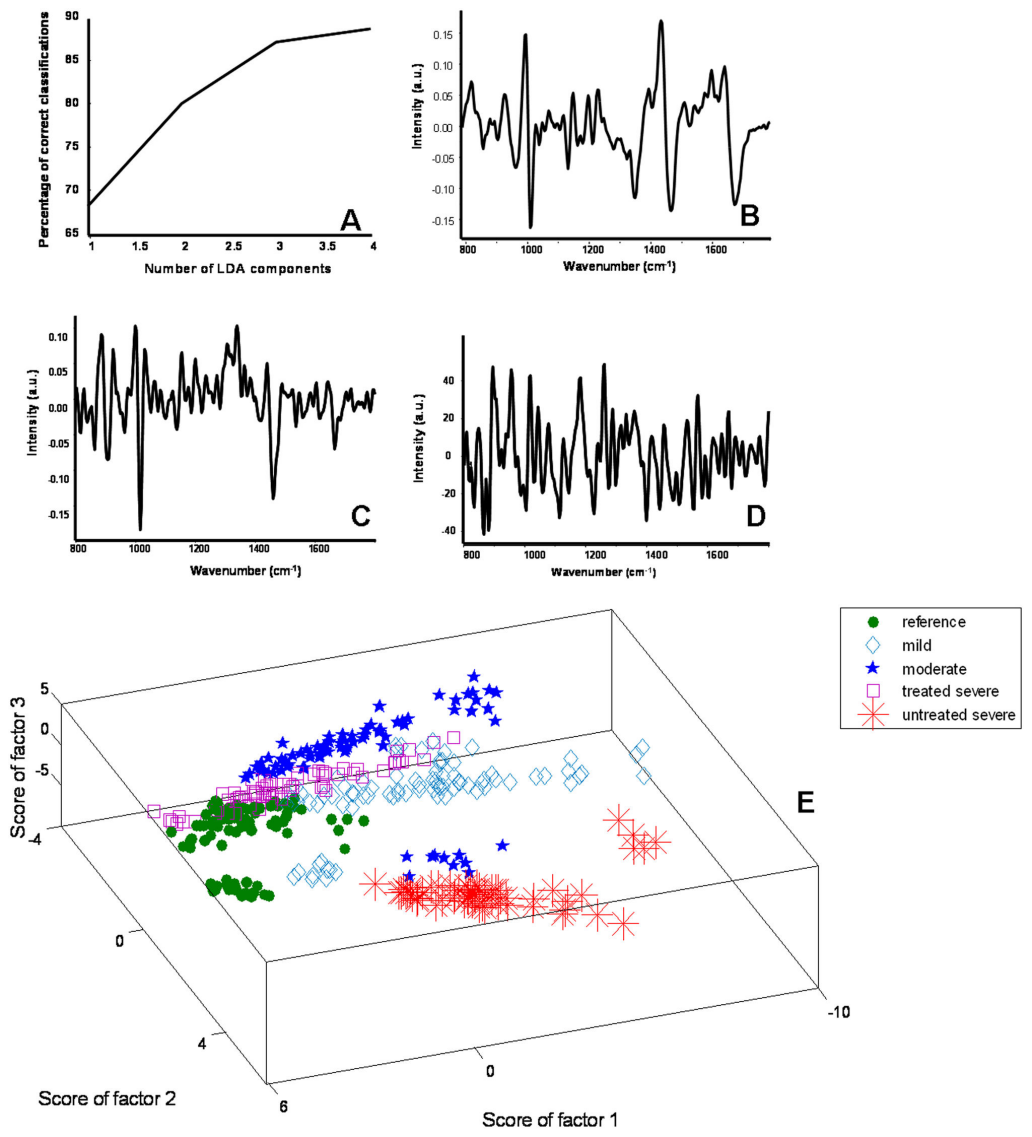
PCA for classification of control and asthma groups. A. Total % variance plot, B. Loadings of factor 1, C. Loadings of factor 2, D. Loadings of factor 3, E. 3D scatter plot.

In the first step, 396 spectra belonging to reference and different asthma grades were subjected to PCA. PCA is an unsupervised classification methodology which explores patterns in the dataset. It decomposes spectral data into small number of independent variations known as factors and contributions of these factors to each spectrum are called scores. The total percent variance plot and loadings of first three factors are shown in Figure 6A-D. Scores of factors 1, 2 and 3 were used to visualize classification between the groups. As shown in Figure 6E, distinct and minimally overlapping clusters were obtained for the groups: reference, mild, moderate, treated severe, while an exclusive cluster for untreated severe was observed. A slight overlap was observed between reference and mild groups. Since PCA is often used as a data overview tool which helps in identifying outliers, groups and trends in the data; supervised classification method PC-LDA was also explored.

**Figure 7:**
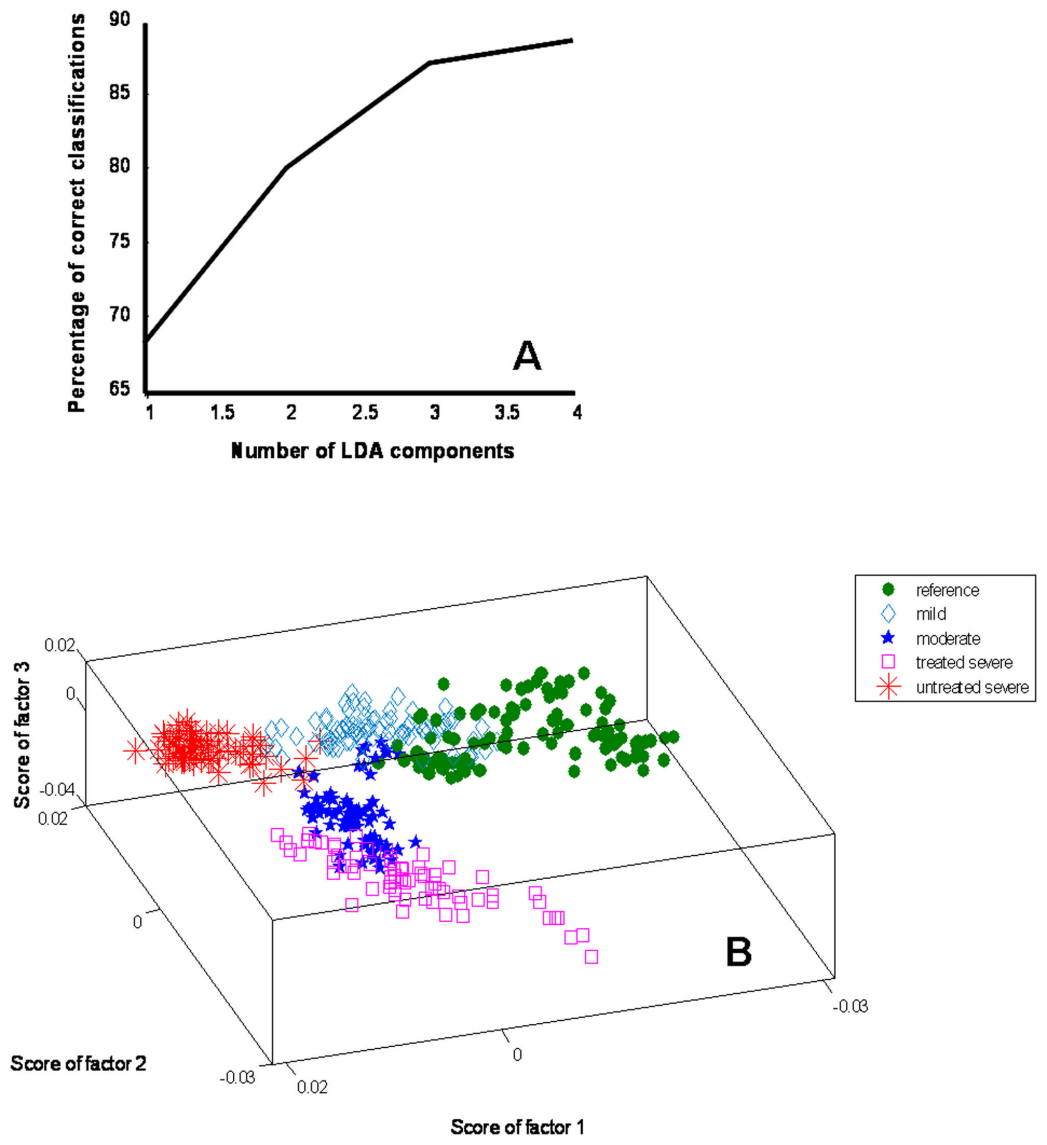
LDA for classification of control and asthma groups. A. Scree plot, B. 3D Scatter plot.

LDA can be used along with PCA (PC-LDA) to further increase the performance efficiency of classification. The advantage of doing this is to remove or minimize noise from the data and concentrate on variables important for classification. To avoid over-fitting of the model, as a thumb rule, total number of factors selected for analysis were less than half the number of the spectra in the smallest group. Four factors contributing ~89% variance were employed for analysis (Figure 7A). The scatter plot was constructed using the scores of first 3 factors shown in Figure 7B indicates mostly well separated clusters for each group, with slight overlap seen between reference and mild asthma, moderate and treated severe asthma groups, while an exclusive cluster for untreated severe cases was observed. This finding is similar to that observed in PCA. The LDA confusion matrix indicates 79 out of 98 reference spectra were correctly classified, where 19 spectra misclassified as mild. In case of mild asthma, 77 spectra were correctly classified, while 2 and 3 spectra misclassified as reference and moderate, respectively. In case of moderate asthma, LDA yielded 70 correct classifications and 10 misclassifications as mild asthma. For treated severe cases, 64 spectra were correctly identified, and 6 spectra incorrectly assigned to moderate groups. In case of the untreated severe group, 61 spectra were correctly classified as untreated severe and 5 spectra misclassified as mild. The LDA results are summarized in Table 2.

**Table 2:** Confusion matrix for LDA of reference, mild, moderate, treated severe and untreated severe age groups.

	**Reference**	**Mild**	**Moderate**	**Treated Severe**	**Untreated Severe**
**Reference**	**79**	19	0	0	0
**Mild**	2	**77**	3	0	0
**Moderate**	0	10	**70**	0	0
**Treated Severe**	0	0	6	**64**	0
**Untreated Severe**	0	5	0	0	**61**

(Diagonal elements are true positive predictions and Ex-diagonal elements are false positive predictions)

LOOCV was carried out to evaluate the results obtained by PC-LDA. Cross-validation also called as rotation estimation, is a technique for assessing performance of a predictive model with a hypothetical validation set when an explicit validation set is not available. As the name suggests leave-one-out (LOO) involves using a single observation from the original sample as the validation data, and the remaining observations as the training data. This is repeated such that each observation in the sample is used once as the validation data and results are averaged over the rounds. The performance is estimated as the number of correct predictions over all the samples used in the data set. In the reference group, 79/98 spectra were correctly classified, while remaining (19.4%) misclassified with mild asthma group. In the mild asthma group, 77 out of 82 spectra were correctly classified as mild, while 2 and 3 spectra were misclassified as reference and moderate asthma, respectively. In the moderate asthma group, 70 spectra were correctly classified, and 10 spectra misclassified as mild asthma. In the treated severe group, 63 spectra were classified as treated severe, and 7 spectra misclassified as moderate asthma. In the untreated group, 61/66 spectra were correctly classified while 5 misclassified as mild asthma. The LOOCV results are summarized in Table 3.

**Table 3:** Confusion matrix for Leave-one-out cross validation of reference, mild, moderate, treated severe and untreated severe age groups.

	**Reference**	**Mild**	**Moderate**	**Treated Severe**	**Untreated Severe**
**Reference**	**79**	19	0	0	0
**Mild**	2	**77**	3	0	0
**Moderate**	0	10	**70**	0	0
**Treated Severe**	0	0	7	**63**	0
**Untreated Severe**	0	5	0	0	**61**

(Diagonal elements are true positive predictions and Ex-diagonal elements are false positive predictions)

Misclassifications of reference with mild asthma group and vice versa could be explained on the basis of less number of biochemical and immunological changes in the serum of mild group, the resemblance evident in the form of misclassifications across the two groups. In the mild group, some misclassifications were also observed with moderate asthma group and most misclassifications of moderate group were with mild group. This could be due to variations in cytokine and chemokine levels in blood, as very little is known about the dynamics of circulating cytokines, including degradation, regulation and half life time. In the treated severe group, most misclassifications were observed with the moderate group. This could possibly be due to reduction in the severity of asthma which had been brought about by the administered pharmacological treatment prior to sample collection. Misclassifications of the untreated severe group were observed only with mild group. These findings are also corroborated by estimation of serum YKL-40 level, where these levels in asthma group (N=44) were found to be significantly elevated with respect to controls, irrespective of the conditions of asthma. However, as the recorded Raman spectra are representative of the entire biochemical composition of the sample, no specific signals related to YKL-40 were observed to establish any correlation. 

Also, no misclassifications of treated severe with untreated severe groups were observed. A statistically insignificant difference between YKL-40 protein levels of treated severe group and reference group was also observed (Table 1 and Figure 2). This may indicate the role of the administered treatment in alleviating the asthmatic symptoms which were simultaneously reflected as changes in serum and were also detected by Raman spectroscopy. This finding has important implications in future, especially in the context of predicting response to treatment and prognosis of the disease. 

## Conclusions

The present study was carried out to explore the feasibility of detecting asthma by RS of sera. Preliminary findings indicated the possibility of classifying reference and asthma conditions distinctly, as well as specific classifications based on the state of severity of the asthmatic grades. Significant elevation in serum YKL-40 levels in asthmatic conditions was also observed. Differences between the groups could also be traced in the spectra of different groups. Spectral comparisons indicate changes in protein structure, increased DNA and possible presence of molecules like histamine, prostaglandins, leukotrienes and glycosaminoglycans (GAGs). These molecules are known to play a role in the inflammatory response, and increased secretion and deposition of total GAGs are seen during asthma. Treated and untreated severe groups were classified distinctly, thus indicating that treatment related changes may also be detected using RS. The limitations of this study were small sample size which comprised of only North Indian asthma patients. As asthma is a heterogenous disease reflecting several phenotypes with varied inflammatory patterns, more studies are warranted on larger sample size by including subjects from different regions of same ethnic group. Treatment related changes observed in this study will also have to be confirmed on more number of subjects. Further, subjects with other pulmonary disorders like, chronic obstructive pulmonary disorder, cystic fibrosis, sarcoidosis, tuberculosis and bronchiectasis would be included to determine disease specificity of the stated method. Prospectively, validating the results of this pilot study using additional blinded studies on a multi-dimensional and large scale may enable routine clinical applications of this methodology for quick, economic and ‘distance’ diagnosis and prognosis of asthma.
